# Cancer Treatment-Induced Accelerated Aging in Cancer Survivors: Biology and Assessment

**DOI:** 10.3390/cancers13030427

**Published:** 2021-01-23

**Authors:** Shuo Wang, Anna Prizment, Bharat Thyagarajan, Anne Blaes

**Affiliations:** 1 Division of Epidemiology and Community Health, School of Public Health, University of Minnesota, Minneapolis, MN 55455, USA; 2 Division of Hematology, Oncology and Transplantation, Medical School, University of Minnesota, Minneapolis, MN 55455, USA; prizm001@umn.edu (A.P.); blaes004@umn.edu (A.B.); 3 Masonic Cancer Center, University of Minnesota, Minneapolis, MN 55455, USA; thya0003@umn.edu; 4 Department of Laboratory Medicine and Pathology, Medical School, University of Minnesota, Minneapolis, MN 55455, USA

**Keywords:** accelerated aging, cancer treatment, cellular senescence

## Abstract

**Simple Summary:**

Many modalities used to treat or control cancer lead to accelerated aging in cancer survivors. However, the effects of cancer treatments on aging in individuals with cancer remain poorly studied. In this review, we summarize the possible biological mechanisms of accelerated aging in cancer survivors induced by cancer treatments. We also discuss the importance of estimating biological age in individuals with cancer and review the methods that can be used to estimate biological age in cancer survivors.

**Abstract:**

Rapid improvements in cancer survival led to the realization that many modalities used to treat or control cancer may cause accelerated aging in cancer survivors. Clinically, “accelerated aging” phenotypes in cancer survivors include secondary cancers, frailty, chronic organ dysfunction, and cognitive impairment, all of which can impact long-term health and quality of life in cancer survivors. The treatment-induced accelerated aging in cancer survivors could be explained by telomere attrition, cellular senescence, stem cell exhaustion, DNA damage, and epigenetic alterations. Several aging clocks and biomarkers of aging have been proposed to be potentially useful in estimating biological age, which can provide specific information about how old an individual is biologically independent of chronological age. Measuring biological age in cancer survivors may be important for two reasons. First, it can better predict the risk of cancer treatment-related comorbidities than chronological age. Second, biological age may provide additional value in evaluating the effects of treatments and personalizing cancer therapies to maximize efficacy of treatment. A deeper understanding of treatment-induced accelerated aging in individuals with cancer may lead to novel strategies that reduce the accelerated aging and improve the quality of life in cancer survivors.

## 1. Introduction

Individuals with and without cancer can age very differently from one another. People with cancer seem to age very rapidly, may appear frail due to cancer treatment, and need assistance in daily routines at age 70, whereas individuals without cancer may not need assistance even at ages much older than 70 [1]. Growing evidence demonstrates that individuals with cancer age faster, so their biological age appears to be older than their chronological age (so-called accelerated aging). According to the definition proposed by Baker and Sprott [2], biological age is characterized by the biological parameter[s] of an organism, either alone or in some multivariate composite. Clinically, “accelerated aging” phenotypes in cancer survivors are characterized by the development of age-related health conditions, including premature mortality and comorbidities – secondary cancers, frailty, chronic organ dysfunction, and cognitive impairment which can impact long-term health and quality of life in cancer survivors. Unfortunately, many therapies used to treat or control cancer may lead to unintended consequences that appear to accelerate aging process [3,4,5]. Cancer treatments can lead to accelerated aging by inciting hallmarks of aging, including telomere attrition, stem cell exhaustion, cellular senescence, DNA damage, and epigenetic alterations [6,7].

This review focuses on the cancer treatment-induced accelerated aging in cancer survivors and approaches to measure the accelerated aging.

## 2. Cancer Treatment-Induced Accelerated Aging in Cancer Survivors

**Figure 1:**
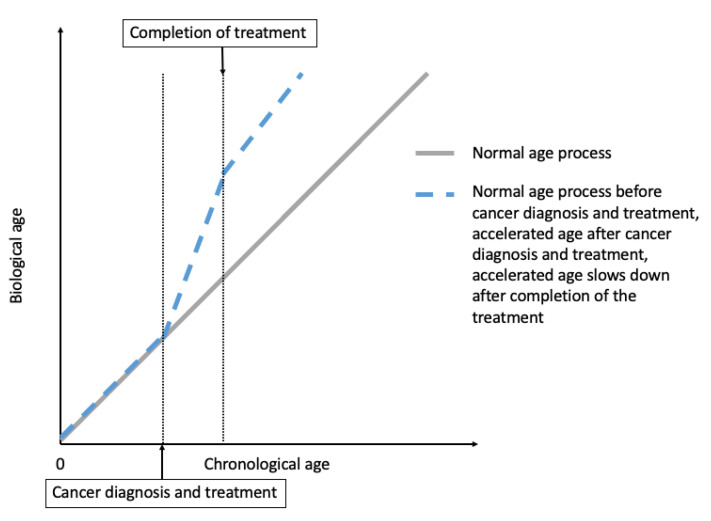
Hypothesized accelerated aging due to cancer treatments, adapted with permission from Guida et al. (2019) [9].

It is not uncommon in clinical practice to observe that cancer patients receiving treatments appear to become “older”. For example, as discussed in Hill et al. [8], a healthy 65-year-old female who “appears to be age 65” is diagnosed with locally advanced breast cancer and receives doxorubicin, cyclophosphamide, and paclitaxel. By the time she completes treatment, she becomes frail and “appears older than age 65” [8]. After stopping the treatment, the acceleration of aging may slow down, sometimes may even reverse, but this patient may not “appear to be age 65” like how she appears to be at the pretreatment level (Figure 1) [8].

### 2.1. Epidemiological Evidence for Treatment-Induced Accelerated Aging

A growing body of literature is pointing toward the effects of cancer treatments on accelerated aging in cancer survivors.

#### 2.1.1. Increased Frailty in Cancer Survivors

There is no one “tool” that is used in the clinical setting to assess frailty, but rather a variety of measures encompassing physical strength, nutritional status, cognition, and physical performance. In research, accelerated aging in cancer survivors has been captured by using some of these measurements such as grip strength, the timed up-and-go (TUG), and the 6-min walk (6MW) test [10]. Lintermans et al. conducted two studies comparing the grip strength after 6 months and 12 months of therapy to the baseline in women with breast cancer receiving aromatase inhibitors, and both studies found a decrease in grip strength after receiving treatment [11,12]. For example, after 6 months of therapy, they found that the grip strength decreased by 8% and 11% for left hand and right hand, respectively (*p* = 0.009) [11]. Furthermore, the Childhood Cancer Survivor Study (CCSS) assessed physical function in young childhood cancer survivors (*n* = 183 cancer survivors, who survived for at least five years; mean age (SD) = 13.5 (2.5)) and their age- and sex-matched siblings (*n* = 147; mean age (SD) = 13.4 (2.4)). This study showed that cancer survivors performed worse than their matched siblings on TUG (*p* = 0.003), and 6 MW (*p* = 0.002), but not grip strength tests (*p* = 0.49) [10]. In agreement with the results from the CCSS, the Rabin Medical Center study (*n* = 26) also reported a worse performance of 6MW test in allogeneic hematopoietic cell transplantation (HCT) survivors compared to their age-matched healthy controls (*p* < 0.005). That study also found no difference in hand grip in HCT survivors and their age-matched controls [13].

Another example stems from studies of hematopoietic cell transplantation (HCT), which reported an increased frailty in cancer survivors [14]. The Bone Marrow Transplant Survivor study (BMTSS) compared risk of frailty in 998 young adult HCT survivors (ages 18–64), who had received HCT for hematologic malignant diseases or severe aplastic anemia and survived at least two years after HCT, and 297 matched siblings (ages 18–64). The study reported that HCT recipients had a higher prevalence of frailty (8.4%) compared to their matched siblings (0.2%) (*p*< 0.001) [14]. The high prevalence of frailty in HCT recipients may be related to their exposure to the high-intensity chemotherapy, radiation, and immunosuppressive agents before, during, and after HCT. These high intensity treatments and complications injure normal tissue and may increase the risk of frailty after HCT, even among non-geriatric HCT patients [14].

#### 2.1.2. Increased Risk of Comorbidities and Premature Mortality in Cancer Survivors

Previous studies suggested that cancer treatments led to accelerated aging that manifested as an increased risk of secondary neoplasms in cancer survivors. For example, another CCSS study of 14,359 childhood cancer survivors, who survived for at least five years (age range: 5–56 years) reported that, after childhood cancer diagnosis (maximum follow-up was 30 years), the cumulative cancer incidence of cancer was 20.5% (95% confidence interval (CI): 19.1–21.8%) for first occurrence of subsequent neoplasms and 7.9% (95% CI: 7.2–8.5%) for second malignant neoplasms (excluding non-melanoma skin cancer). The greatest cumulative incidence was among people surviving Hodgkin lymphoma (HL). This study also found a 170% (95% CI: 2.2–3.3) higher risk of all first subsequent neoplasms in cancer survivors who had been exposed to radiation therapy for their primary cancer diagnosed before 21 years of age compared to those who had not been exposed to radiation therapy [15]. Results from this study suggested that cancer survivors were at a higher risk of developing secondary neoplasm, and diagnosis of HL and treatment with radiation therapy were associated with an increased risk of secondary neoplasm [15].

Other studies reported an increased risk of cardiovascular diseases (CVDs) in both childhood and adult cancer survivors after treatment. For example, the St. Jude lifetime cohort study compared the cumulative incidence CVDs in 670 pediatric, adolescent, or young adult HL survivors with their age- and sex-matched controls from general population (*n* = 270), who were selected regardless of past medical history [16]. That study showed that the incidence of at least one adverse event of grade 3–5 involving chronic cardiovascular health conditions by age 50 was 45.5% (95% CI: 36.6%–54.3%) for the HL survivors, who had been treated with chemotherapeutic agents and radiation dosimetry compared to a cumulative incidence of 15.7% (95% CI: 7.0%–24.4%) in their matched controls [16]. Furthermore, the Kaiser Permanente Southern California study (KPSC) (36,232 over two-year adult cancer survivors and 73,545 matched cancer-free KPSC members) reported a higher incidence rate of CVDs in breast (incidence rate ratio (IRR)= 1.13, 95% CI= 1.06–1.22), kidney (IRR = 1.24, 95%CI: 1.02–1.51), lung (IRR = 1.58, 95% CI: 1.30–1.90), and ovary (IRR= 1.41, 95% CI: 1.06–1.88) cancer survivors compared to their age-, sex-, and residual zip code-matched cancer-free controls [17]. This study also reported a 11% decreased incidence rate of CVDs in prostate cancer survivors compared to their matched controls (95% CI: 0.84–0.95). However, that study found no difference in incidence rate of CVD in overall cancer survivors, and survivors of bladder, chronic lymphocytic leukemia, colon, rectal, thyroid, and uterus cancers compared to their matched controls [17]. Another example stems from the UK electronic health records databases study [18]. This study reported an increased risk of venous thromboembolism in cancer survivors of colorectum, lung, breast, uterus, prostate, and non-HL who were treated with chemotherapy compared to survivors of these cancers who did not receive chemotherapy [18].

Another CCSS study (*n* = 6,148) assessed the increased risk of late mortality (more than five years after diagnosis) in long-term survivors of childhood acute lymphoblastic leukemia (ALL) as a result of their cancer treatment [19]. With a maximum of 20 years follow-up, the overall late mortality rate was 360% (95% CI: 4.2–5.1) higher than the rate in their age-, sex-, and race-matched US population [19]. Furthermore, Yeh et al. developed a model to estimate the life expectancy for a cohort of 15-year-old five years ALL survivors [20]. The estimated life expectancy for this ALL survivors was 50.6 years, which equals to a loss of 10.4 years compared to the general population [20].

Literature pointing to the accelerated aging in people with cancer syndromes is scarce. Previous studies hypothesized that people with Li Fraumeni syndrome (LFS) were in the trajectory of accelerated aging, as people with LFS were at a greater risk of developing cancer [21]. In addition, results from studies showed that people with hereditary syndromes like Fanconi anemia were more susceptible to damage from cancer therapies such as radiation treatment [22].

These data collectively demonstrated that cancer survivors had lower physical function, higher risks of premature death, and age-associated morbidities such as secondary neoplasms and CVDs, suggesting an accelerated aging process after treatment.

### 2.2. Biological Mechanisms Underlying Treatment-Induced Accelerated Aging in Cancer Survivors

**Figure 2:**
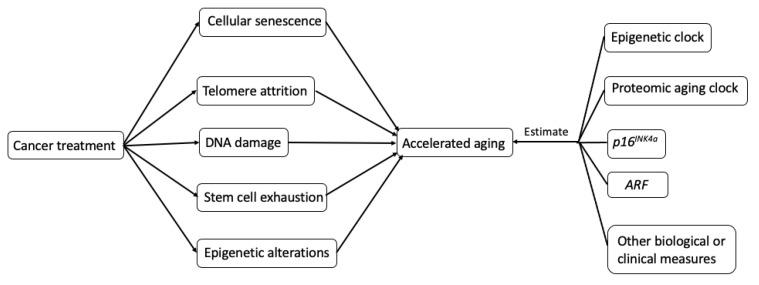
Possible biological mechanisms and potential measurements of treatment-induced accelerating aging in cancer survivors.

The potential biological mechanisms (Figure 2.) that explain how cancer treatments may incite hallmarks of aging (telomere attrition, stem cell exhaustion, cellular senescence, DNA damage, and epigenetic alterations) are shown in Figure 2 [6].

#### 2.2.1. Cellular Senescence and SASP

##### Cellular Senescence and Aging

Cellular senescence is defined as an irreversible arrest of cell proliferation. Cellular senescence was hypothesized to be a tumor-suppressing mechanism [23]. Based on the hypothesis that cancer cells can proliferate indefinitely, cellular senescence can preclude cancer cells from proliferating. Since it may preclude cancer cells from proliferating, cellular senescence has been seen as a powerful strategy for cancer treatment. Many commonly used cancer treatments—e.g., chemotherapy, radiotherapy, CDK4/6 inhibitors, epigenetic modulators, and immunotherapy—intend to induce senescence in tumor cells. However, the same therapies also cause cellular senescence in neighboring non-tumor tissues [24]. Also, cellular senescence is often accompanied by a complex pro-inflammatory response—senescence-associated secretory phenotype (SASP). SASP is characterized by the secretion of SASP factors including numerous proinflammatory cytokines (e.g., interleukin-6 (IL-6) and IL-8), chemokines (e.g., monocyte chemoattractant proteins (MCPs) and macrophage inflammatory proteins (MIPs)), growth factors (e.g., transforming growth factor-β (TGFβ) and vascular endothelial growth factor (VEGF)), and proteases [25,26,27,28,29,30]. The secretion of those SASP factors can lead to accelerated aging in cancer survivors by triggering inflammation, promoting tumorigenesis and development of age-associated diseases, e.g., cognitive impairment [31] and CVD [32]. In this review, we focus on the mechanism of SASP promoting tumorigenesis.

##### SASP and Tumorigenesis

SASP factors can promote cancer cell proliferation, migration, invasiveness, angiogenesis, and epithelial-mesenchymal transition (EMT) [24]. Here, we briefly review the effect of SASP on each of these processes.

Evidence showed that SASP factors could promote cancer cell proliferation. For example, in the immunocompromised (nu/nu) mice model, tumors developed in seven out of 15 animals injected with senescent fibroblasts, while no tumors developed in eight animals injected with non-senescent fibroblasts [33]. One of the possible mechanisms is one of the SASP factors—IL-6—promotes tumor progression. IL-6 can promote proliferation by binding to the IL-6 receptor and subsequent activation of STAT3. STAT3 is widely considered as oncogene [34,35], and it can promote cancer progression through the transcription target genes [36].

SASP factors could promote cancer cell migration. For example, breast cancer cells showed the ability to migrate when induced with senescent fibroblasts [29]. This ability to migrate may be promoted by the secretion of SASP factors, e.g., IL-6 and IL-8, which can mediate the activation of STAT3.

SASP factors can promote EMT, an important step in cancer progression that allows the solid tumors to become more malignant, increasing their invasiveness and metastatic activity [37,38]. Coopé et al. incubated non-aggressive human breast cancer cell lines (T47D and ZR75.1) with conditioned media from senescent fibroblasts or pre-senescent fibroblasts induced by X-irradiation [29]. They found that fibroblast SASP induced a classic EMT in T47D and ZR75.1 cells [29]. Conditioned media from senescent cells markedly decreased overall and cell surface β-catenin and E-cadherin, and reduced cytokeratin expression, consistent with a mesenchymal transition. Furthermore, conditioned media from senescent cells downregulated the tight junction protein claudin-1, leaving the remaining protein localized primarily to the nucleus, a hallmark of an EMT [29]. IL-6 is likely to be one of the SASP factors that involved in the process of promoting EMT. Findings from the in vitro study showed IL-6 could induce EMT through the activation of STAT3 in human cervical carcinoma cells [39].

VEGF, one of the SASP factors, can promote tumor-driven angiogenesis, which is one of the hallmarks of cancer [40,41]. For example, in two groups of mice, a larger number and bigger sizes of vessels were observed in the group that was additionally injected with senescent mouse breast fibroblasts compared to the group that was injected with EpH4-v epithelial cells alone [40].

Although many commonly used cancer interventions induce senescence in tumor cells, SASP, a complex pro-inflammatory response of senescent cells, can lead to accelerated aging in cancer survivors by promoting various aspects of tumorigenesis and age-associated diseases.

#### 2.2.2. Telomere Attrition

Telomeres are protein-bound DNA repeated structures at the ends of chromosomes that cap and stabilize the ends of chromosomes [42]. Telomeres shorten during each cycle of cellular division until the cell reaches its limited ability to divide. Telomerase, the enzyme, protects the telomere by maintaining telomere length in human cells [43,44]. As it decreases with age, telomere length has been proposed as a biomarker of aging [42,43,44].

Many cancer treatments impact telomere length, and some directly impair telomerase [45,46]. For example, chemotherapy can cause telomere shortening. It is not uncommon to see telomere length shortening among cancer survivors after receiving chemotherapy. Results from a study of 15 people with non-HL receiving conventional-dose chemotherapy showed that the mean telomere length decreased after chemotherapy (mean telomere length: 7.59 kb (before chemotherapy) vs. 7.07 kb (after chemotherapy), *p* = 0.03). The mean of their telomere length after chemotherapy was shorter compared to the mean of telomere length in their age-matched putatively cancer-free controls (mean telomere length: 8.71 kb (health controls) vs. 7.07 kb (people with cancer after chemotherapy), *p* < 0.01) [47]. In a study of 25 children with pediatric acute leukemia (*n* = 16) or solid tumor (*n* = 9), Engelhardt et al. found that the telomere length shortened in children after receiving chemotherapy [48]. This finding is in line with the laboratory studies of cancer cells line. In the study of BEL-7404 human hepatoma cell line, Zhang et al. found that the mean telomere lengths were decreased after the chemotherapy treatment (mean telomere length ranged from 3.1 to 4.1 kbp in the treated cells vs. 4.2 kbp of controls) [45].

In summary, many pieces of evidence indicated that cancer treatments could lead to a shorter telomere length, suggesting accelerated aging in cancer survivors.

#### 2.2.3. Stem Cell Exhaustion

Stem cell exhaustion is the age-related reduction in activity of stem cells. Stem cells perform a wide range of functions, including the replacement of damaged or lost red blood cells and white blood cells [49,50]. In normal aging, the activity of stem cells decreases with age. For example, the National Marrow Donor Program (MNDP) study found that donor age was associated with lower overall and disease-free survival in bone marrow transplant recipients, suggesting that the activity of hematopoietic stem cells (HSCs) diminishes with age [51,52]. The stem cells may undergo an accelerated aging process. For example, after HCT, HSCs undergo replicative stress to allow hematopoietic reconstitution [53]. The replicative stress of HSCs occurring after HCT may lead to accelerated aging, as it can cause premature bone marrow failure, which is characterized by an inability to make enough blood cells, including red blood cells and white blood cells, in patients [54,55]. A single transplantation of HSCs was shown to effectively double the cellular aging that would occur normally [56].

#### 2.2.4. DNA Damage

DNA damage, a critical factor in cancer development and progression, can also contribute to aging [57,58]. The DNA damage can be measured by γH2AX, which accumulates at the site of DNA damage and can be detected by immunocytochemistry [59,60]. Direct damage to DNA can be caused by the free radical intermediates generated by chemotherapeutic agents [61]. The alkylating agents, the oldest class of anticancer drugs, can lead to DNA double-strand breaks [57], while the DNA damage caused by alkylating agents is also associated with an increased risk of developing secondary leukemias in patients who treated with these agents [62,63]. For example, secondary leukemia has been reported in both children and older adults previously treated with alkylating agents [64,65]. Besides alkylating agents, cytotoxic drugs and ionizing radiation can also cause DNA damage [66].

#### 2.2.5. Epigenetic Alterations

Many epigenetic alterations that are induced by cancer treatment drugs are likely to trigger the accelerated aging process. The well-known epigenetic modification, CpG island hypermethylation, is a component of drug-induced cytotoxicity. CpG island hypermethylation could cause inappropriate silencing of specific genes that may render the genome unstable and contribute to aging [67].

The examples of commonly used chemotherapy agents that can induce DNA hypermethylation at CpG islands in tumor cells include topoisomerase II inhibitors, doxorubicin, DNA cross-linking agents, and methotrexate [68]. For example, the alteration of 5-methylcytidine content was found in human lung epidermoid carcinoma cells after these cells were exposed to topoisomerase II inhibitors [68]. Some other drugs, including azacitidine and decitabine, hydralazine, and MG98, have also been reported as DNA hypermethylation agents. By inducing hypermethylation, all of these drugs may lead to accelerated aging in people with cancer [67].

In summary, these are the possible mechanisms that may explain the cancer treatment-induced accelerated aging in cancer survivors. At this time, most of the literature on accelerated aging from cellular senescence is based on data around the use of cytotoxic chemotherapy agents as well as radiation treatment. Further research is needed to clarify how targeted therapy and immunotherapy may impact cellular senescence.

## 3. Measurements of Accelerated Aging in Cancer Survivors

To estimate accelerated aging in cancer patients, researchers introduce the term called biological age that could better predict the aging process than chronological age. For cancer survivors, the biological age is expected to predict the risk of health conditions in cancer survivors—e.g., secondary cancers, frailty, chronic organ dysfunction, and cognitive impairment—better than chronological age. Although measuring biological age in this group is very important, only a few studies examined biological aging in cancer survivors, including aging after receiving treatment. Understanding the biological age and intrinsic biological mechanisms of aging can help physicians provide a more individualized cancer therapy during the active treatment and potentially add interventions, such as anti-aging agents. The anti-aging agents (e.g., senolytics [69], resveratrol, and metformin) could potentially reverse aging or at least slow down the accelerated aging process and improve quality of life in cancer survivors during the active treatment and after the treatment ends. This is an important area for further research.

Several clinical and biological measures have been proposed to capture the biological age, including gait speed, grip strength, TUG, 6MW, Fried (Cardiovascular Health Study) Frailty Phenotype, Deficit Accumulation Index/Frailty Index, clinical geriatric assessment, cognitive assessments (e.g., Hopkins Verbal Learning, Controlled Oral Word, the Trail Making Test, etc.), fatigability, APOE4, 31p recovery time, telomere length, chronic inflammatory biomarkers, maximal oxygen consumption, sarcopenia, phenotypic age, allostatic load, biomarkers of cellular senescence, and aging clocks [9,10,42,70,71,72]. In this review, we will focus on three important biomarkers for aging: epigenetic clock, proteomic aging clock, and two critical biomarkers of cellular senescence—p16^IKN4a^ and ARF (an alternate reading frame protein product of the CDKN2A locus, also known as P14^ARF^ in humans and P19^ARF^ in mice) (Figure 2).

### 3.1. Aging Clocks

Aging clocks are a set of molecules that are being measured in blood or multiple tissues and are capable of predicting individuals’ biological age independent of their chronological age. Several aging clocks have been developed, including epigenetic clock and proteomic aging clock.

#### 3.1.1. Epigenetic Clock

Epigenetic clock, a set of DNA methylation-based biomarkers in blood or tissue, is the most acknowledged among all the aging clocks [73,74]. Among all the epigenetic clocks, the Horvath and Hannum epigenetic clocks are the oldest and the most popular predictors of chronological age. Horvath’s clock is calculated based on methylation levels of 353 CpGs sites obtained from a variety of tissues and cell types. The Hannum clock is derived from only 71 CpGs sites using whole blood samples. Both of these clocks show high correlations with chronological age (r = 0.96 for Horvath and r = 0.91 for Hannum) and small mean differences from chronological age (3.6 for Horvath and 4.9 years for Hannum) in their corresponding validation sets [73,74]. Besides these two clocks, Levine et al. developed the DNAm PhenoAge based on 513 CpGs from whole blood and found a correlation with chronological age of 0.94 in their validation set [75]. The important difference between DNAm PhenoAge and the Horvath and Hannum clocks is that the DNAm PhenoAge was developed using markers correlated with phenotypic age, not chronological age. Therefore, DNAm PhenoAge was hypothesized to be a more powerful predictor of age-related diseases [75]. Moreover, Lu et al. developed the DNAm GrimAge based on 1030 CpGs with correlations of chronological age of 0.79–0.95 in different validation cohorts [76]. The importance of this epigenetic clock is that it is a composite biomarker based on the seven DNAm surrogates and a DNAm-based estimator of smoking pack-years, which found to be a better predictor of mortality than the actual observed biomarkers [76]. Using blood samples, a recent study developed another novel epigenetic clock—DeepMage, which showed a mean difference from chronological age of 2.77 in the validation set of healthy individuals [77]. The difference between DeepMage and all other clocks is that DeepMage was developed using a deep learning approach, not relying on linear regression method [77]. The DeepMage showed biological relevance by assigning a higher predicted biological age to people with various health conditions. For example, they compared the average prediction errors in a case-control study design. Women with ovarian cancer were predicted to be 1.7 years older than the controls (average prediction errors: cases = −1.27 and controls = −2.97, *p* < 0.05) [77]. Other epigenetic clocks include Weidner’s [78], ELOVL2, and FHL2 clocks [79].

Epigenetic clocks have been applied in epidemiological studies to predict the risks of different diseases and cancers. Several studies have used Horvath and Hannum clocks, DNAm PhenoAge, and DNAm GrimAge to predict the risk of cancer, including overall cancer and cancers of lung, breast, colorectum, and pancreas [75,80,81,82,83,84]. In the meta-analysis of seven cohort, Lu et al. found a statistically significantly association between DNAm GrimAge acceleration (GEAA) and risk of overall cancer (HR= 1.07, 95% CI: 1.05–1.08) [76]. In the U.S. Department of Veterans Affairs’ Normative Aging Study (*n* = 442), Zheng et al. estimated epigenetic age as measured by Hannum’s 71-CpG method using Horvath’s online calculator. They found that each one unit increase in their epigenetic age acceleration was associated with a 6% increased risk of developing any cancer within three years (95% CI: 1.02–1.10) [84]. In the Women’s Health Initiative (WHI) study (*n* = 2029), Levine et al. found that a one-year increase in Horvath clock [80] was statistically significantly associated with a 50% (*p* = 3.4 × 10^−3^) increase in the risk of lung cancer [80], and in another WHI study, Levine et al. found that a one-year increase in DNAm PhenoAge was statistically significantly associated with a 5% (*p* = 0.031) increase in the risk of lung cancer [75]. In the Sister Study (*n* = 2764), Hannum age acceleration (EEAA), Horvath age acceleration (IEAA), and DNAm PhenoAge age acceleration (PEAA) were statistically significantly associated with increased breast cancer risk with the strongest association observed for PEAA: HR (95% CI) = 1.15 (1.07, 1.23) [82]. Previous findings were inconsistent regarding colorectal cancer and pancreatic cancer risk when using different epigenetic clocks [81,83,85]. For instance, the EPIC-Italy study (*n* = 845) found that male had an increased colorectal cancer (CRC) risk associated with Horvath clock (*p* = 0.042) and FHL2 (*p* = 0.036) clocks, but not Hannum, Weidner, or ELOV2 [83]. However, for female, they found they found no associations between any of the five clocks and CRC risk [83]. A pooled analysis of Nurses’ Health Study, Physician’s Health Study, and the Health Professionals follow-up study (*n* = 824) found positive dose-response trends of IEAA and PEAA with pancreatic cancer risk with a stronger association observed for Hannum clock: Q4 vs. Q1: OR (95% CI) = 1.73 (1.11, 2.71), however, for Horvath age acceleration, the highest OR was found in the third quartile compared to the lowest quartile [81].

Not only has epigenetic clock been used to predict cancer risk, but also it has been tested in cancer survivors. For example, a recent study of 72 women with early-stage breast cancer (37 received radiation therapy alone and 35 received chemotherapy and radiotherapy) reported a statistically significantly increase in EEAA (*p* = 0.0021), PEAA (*p* = 0.015), and GEAA (*p* = 3.2 × 10^−6^) from pre-treatment to post-treatment, but not in IEAA [86]. Another study tested the Weidner’s clock in 19 patients with HCT [13]. That study reported a higher value of the median of Weidner’s clock compared to the median of chronological age in patients with HCT (*p* < 0.04) [13].

In summary, several types of epigenetic clocks have been developed using multi-tissue or whole blood. All of those clocks showed high correlations with chronological age. Some clocks were associated with risk of different cancer types, but the magnitude of associations depends on the type of clock used and type of cancer in the study. It is possible that inconsistencies may be partially explained by the use of different types of clocks and the small sample size of the studies. Although measuring biological age in cancer survivors is very important, to our knowledge, only a few studies tested these epigenetic clocks in cancer survivors in epidemiological studies. In addition, validations are needed before these epigenetic clocks can be widely used in clinics.

#### 3.1.2. Proteomic Aging Clock

Besides epigenetic clock, the proteomic aging clock, which combines a set of proteomic-based aging-related biomarkers in the blood, has been proposed to be a potential biological age estimator. These protein biomarkers may be promising markers of aging because they, as intermediate phenotype, can reveal direct information on biological pathways that are involved in many physiological and pathological manifestations of aging [87,88]. In a systematic review of 36 studies, Johnson et al. proposed two versions of proteomic aging clock: the 23-protein panel and the 83-protein panel. They developed and evaluated the prediction accuracy of these two panels using the INTERVAL cohort, which comprised 3301 healthy individuals aged 18–76 years, as the training (two-thirds of samples in the INTERVAL cohort) and validation sets (one-third of the samples in the INTERVAL cohort) [89]. In their validation set, the 23-protein and 83-protein panel had Pearson correlations of 0.66 and 0.87 with chronological age, respectively, and mean absolute differences between the aging clock and chronological age of 8.17 and 4.88 in years, respectively [90]. Additional proteomic aging clocks have been created by Satyan et al. using the LonGenity cohort (*n* = 1025, age range: 65–95 years), which included two groups of offsprings with different survival of parents. One group had parents with exceptional longevity (at least one parent lived to age 95 or older), and the other group had parents with usual survival (having neither parents who lived to age 95) [91]. They created four clocks that consisted of 162, 75, 67, and 35 proteins. The 162-protein clock was selected by elastic net regression from 4265 proteins. While the 75-, 67-, and 35-protein clocks were selected by elastic net regression from the top significant 200, 100, and 50 proteins, in the 4265 proteins, associated with chronological age. The 162-, 75-, 67-, and 35-protein clocks had high correlations with chronological age of 0.79, 0.80, 0.79, and 0.78, respectively [91]. Furthermore, another study of healthy people by Tanaka et al. found correlation of 0.94 between a proteomic aging clock, including 76 proteins, and chronological age [87]. To determine the minimum number of proteins required to create a meaningful proteomic aging clock, Tanaka et al. fitted a series of models. They found that a proteomic age clock that only contained eight proteins could reach a correlation with chronological age of 0.92 [87]. Even higher correlation of 0.97 was observed between a proteomic aging clock including 373 proteins and chronological in the study that combined the INTERVAL and LonGenity cohorts [92]. Thus, since a proteomic aging clock that contained less than ten proteins could reach a correlation with chronological age of more than 0.9, it has a very high precision [87].

In summary, several different proteomic aging clocks have been developed, and all of them showed high correlations with chronological age. When creating proteomic clocks, some studies included only proteins that correlated with age, while others, also considered proteins associated with sex, race, or other variables [87,92]. The next step is to develop and validate a standardized proteomic aging clock that could be widely used in clinics

### 3.2. Biomarkers of Cellular Senescence

Several biomarkers of cellular senescence have been proposed to be potential biomarkers of aging, including p16^INK4a^, ARF, β-galactosidase (SAβ-gal), *Rb* hyperphosphorylation, and SASP components, such as IL-6 and IL-8 [70,93,94]. We chose to discuss the p16^INK4a^ and ARF in more detail because in vitro studies consistently showed the importance of p16^INK4a^ and/or ARF in the senescence of different human cell types, e.g., pancreatic islet cells [95], fibroblasts [96,97], and epithelial cells [98]. In addition, the in vivo studies indicated that p16^INK4a^ and ARF regulated the proliferation and apoptosis of stem cells [99,100,101,102].

#### 3.2.1. p16^INK4a^

p16^INK4a^ is a cell cycle protein whose expression in peripheral blood T lymphocytes increases exponentially with chronological age [103]. In a cohort of healthy donors (aged 18–80), Liu et al. compared the expression of p16^INK4a^ in different cell types of human blood, including T cells (CD3^+^), B cells (CD19^+^), monocytes (CD14^+^), NK cells (CD56^+^), and granulocytes (CD15^+^ or CD16^+^) with the highest expression of p16^INK4a^ found in CD3^+^ T cells [103]. These researchers also found that the expression of p16^INK4a^ in T lymphocytes from peripheral blood appeared to exponentially increase with chronological age in healthy donors. This study proposed that p16^ink4a^ in the peripheral blood T lymphocytes may be a useful peripheral blood biomarker for human biological age as it can be easily measured via low-cost test [103]. In agreement with that finding, other studies found that the expression of p16^INK4a^ increased with age in rodent, baboon, and human tissues [104,105,106,107,108,109,110]. However, to our knowledge, only a few studies examined p16^ink4a^ in cancer survivors or used it to predict the risk of cancer. Sanoff et al. conducted both prospective and cross-sectional analyses among 33 women with stage I and II breast cancer. The results from both analyses showed the expression of p16^INK4a^ increased after receiving chemotherapy [111]. For example, in their prospective analysis, they found that the expression of p16^INK4a^ increased immediately after receiving chemotherapy and remained elevated 12 months after treatment. The absolute increase in the expression was approximately 75%, which is equivalent to the increase observed over 14.7 years of chronological aging [111]. Furthermore, a recent study of allogeneic HCT survivors reported a significantly higher expression of p16^INK4a^ in 19 HCT survivors at a median of 3.75 (range, 2.25–9) years after transplantation compared to their donors (*p* < 0.04) [13]. These findings agree with the results of a recent case-control study (*n* = 32) of testicular cancer survivors treated with chemotherapy [112]. That exploratory study found that in testicular cancer survivors, who were in surveillance and without evidence of disease for more than three months, the expression of p16^INK4a^ was higher than the expression level in their age-matched healthy controls (*p* = 0.048) [113]. Besides those studies, a recent case-control study (352 breast cancer cases and 324 controls) used the expression of p16^INK4a^ in T cells to predict the risk of breast cancer. They found that each 0.1 unit increase in the expression of p16^INK4a^ was associated with a 36% increase in the risk of breast cancer (95% CI: 1.19–1.58) [114].

In summary, p16^INK4a^ may be a useful single blood biomarker as it is easily measurable in a non-invasive way. Validation studies are needed to determine if this biomarker can be applied in clinics.

#### 3.2.2. ARF (An Alternate Reading Frame Protein Product of the CDKN2A Locus)

ARF (also known as P14^ARF^ in humans and P19^ARF^ in mice) is a tumor suppressor protein that has a critical role in the prevention of cancer development through regulating cell proliferation, senescence, and apoptosis [115]. Similar to p16^INK4a^, animal studies found an increase in the expression of ARF in young vs. old tissues in multiple organs, including brain, heart, duodenum, kidney, and uterus [104,110]. In the prospective study of 33 women with stage I and II breast cancer, Sanoff et al. found the expression of ARF increased immediately after receiving chemotherapy and remained elevated 12 months after treatment (*p* < 0.001) [111].

To our knowledge, only a few studies measured ARF in cancer survivors. Additional research is warranted to validate if ARF could be widely used as a biomarker of aging.

## 4. Conclusions

Growing evidence has shown that cancer treatments contribute to accelerated aging in cancer survivors. To capture the accelerated aging, several clinical and biological measures have been proposed to be potential biomarkers of biological age. However, the accelerated aging in cancer survivors remains poorly studied.

Measuring the biological age has a great implication in cancer survivors as it can quantify the accelerated aging during the active treatment and after treatment ends. Measuring biological age may help identify the determinants of the accelerated aging process and better understand the biological mechanism of aging. This knowledge would inform personalizing cancer therapies to maximize the efficacy of treatment and reduce their deleterious effect on aging. Moreover, compared to chronological age, biological age may assist in predicting risks of cancer treatment-related comorbidities, such as frailty and cognitive impairment, which can impact long-term health outcomes and quality of life in cancer survivors. As the importance of measuring biological age in cancer survivors becomes evident, more studies are needed to investigate the aging-related consequences of cancer treatment in cancer survivors.

In brief, future research is warranted to better understand the biological mechanisms of aging. Since anti-aging drugs are available, a deeper understanding of aging-related consequences of cancer and cancer treatments would lead to new strategies to mitigate or even reverse aging and will eventually improve the quality of life of cancer survivors.
